# Prognostic modeling of hepatocellular carcinoma based on T-cell proliferation regulators: a bioinformatics approach

**DOI:** 10.3389/fimmu.2024.1444091

**Published:** 2024-10-09

**Authors:** Long Hai, Xiao-Yang Bai, Xia Luo, Shuai-Wei Liu, Zi-Min Ma, Li-Na Ma, Xiang-Chun Ding

**Affiliations:** ^1^ Department of Infectious Disease, General Hospital of Ningxia Medical University, Yinchuan, Ningxia, China; ^2^ Infectious Disease Clinical Research Center of Ningxia, General Hospital of Ningxia Medical University, Yinchuan, Ningxia, China; ^3^ Weiluo Microbial Pathogens Monitoring Technology Co., Ltd. of Beijing, Beijing, China; ^4^ Department of Tropical Disease & Infectious Disease, The Second Affiliated Hospital of Hainan Medical University, Haikou, Hainan, China

**Keywords:** T-cell proliferation regulators, hepatocellular carcinoma, bioinformatic, GEO, prognostic model

## Abstract

**Background:**

The prognostic value and immune significance of T-cell proliferation regulators (TCRs) in hepatocellular carcinoma (HCC) have not been previously reported. This study aimed to develop a new prognostic model based on TCRs in patients with HCC.

**Method:**

This study used The Cancer Genome Atlas-Liver Hepatocellular Carcinoma (TCGA-LIHC) and International Cancer Genome Consortium-Liver Cancer-Riken, Japan (ICGC-LIRI-JP) datasets along with TCRs. Differentially expressed TCRs (DE-TCRs) were identified by intersecting TCRs and differentially expressed genes between HCC and non-cancerous samples. Prognostic genes were determined using Cox regression analysis and were used to construct a risk model for HCC. Kaplan-Meier survival analysis was performed to assess the difference in survival between high-risk and low-risk groups. Receiver operating characteristic curve was used to assess the validity of risk model, as well as for testing in the ICGC-LIRI-JP dataset. Additionally, independent prognostic factors were identified using multivariate Cox regression analysis and proportional hazards assumption, and they were used to construct a nomogram model. TCGA-LIHC dataset was subjected to tumor microenvironment analysis, drug sensitivity analysis, gene set variation analysis, and immune correlation analysis. The prognostic genes were analyzed using consensus clustering analysis, mutation analysis, copy number variation analysis, gene set enrichment analysis, and molecular prediction analysis.

**Results:**

Among the 18 DE-TCRs, six genes (*DCLRE1B*, *RAN*, *HOMER1*, *ADA*, *CDK1*, and *IL1RN*) could predict the prognosis of HCC. A risk model that can accurately predict HCC prognosis was established based on these genes. An efficient nomogram model was also developed using clinical traits and risk scores. Immune-related analyses revealed that 39 immune checkpoints exhibited differential expression between the high-risk and low-risk groups. The rate of immunotherapy response was low in patients belonging to the high-risk group. Patients with HCC were further divided into cluster 1 and cluster 2 based on prognostic genes. Mutation analysis revealed that *HOMER1* and *CDK1* harbored missense mutations. *DCLRE1B* exhibited an increased copy number, whereas *RAN* exhibited a decreased copy number. The prognostic genes were significantly enriched in tryptophan metabolism pathways.

**Conclusions:**

This bioinformatics analysis identified six TCR genes associated with HCC prognosis that can serve as diagnostic markers and therapeutic targets for HCC.

## 1 Introduction

Hepatocellular carcinoma (HCC), a major health issue, is the sixth most common malignancy and the third most common cause of cancer-related mortality (1). The incidence and mortality rates of HCC are high in Asian and African populations, which account for approximately 75% of new HCC cases worldwide. The annual incidence of new HCC cases is approximately 780,000, with approximately 600,000 deaths worldwide. China accounts for 55% of the global HCC incidence (2). The high HCC incidence in China is closely linked to various factors, such as viral hepatitis, chronic alcohol abuse, obesity, and diabetes (3). As the symptoms are not prominent in the early stages, 70%–80% of patients are diagnosed with advanced-stage HCC (4). Currently, the therapeutic modalities for HCC include liver transplantation, surgical resection, radiofrequency ablation, transarterial chemoembolization (TACE), and targeted drug therapy. The 5-year overall survival (OS) rate of patients with early-stage HCC after radical surgery can be as high as 80%. However, the survival rate of patients with advanced-stage HCC is less than 20% (5–9). Recently, immunotherapy, especially T-cell-related therapies involving immune checkpoint inhibitors (ICIs), has revolutionized the treatment landscape for advanced HCC. However, the overall objective response rate of patients undergoing ICI therapy is low (10, 11), which can be because the role of the immune microenvironment in HCC pathogenesis has not been elucidated.

T cells, including cytotoxic T cells, T helper cells, and T regulatory (Treg) cells, play a crucial role in the tumor microenvironment (12). Expression of inhibitory molecules, such as programmed death ligand 1 (PD-L1), promotes the malignant proliferation of HCC cells. The binding of PD-L1 to its receptor (PD-1) suppresses T-cell activation and proliferation, impedes the ability of immune cells to attack tumors, and promotes malignant growth (13). ICIs, which are a type of immunotherapeutic agents, target the PD-1/PD-L1 pathway in the immune microenvironment and can potentiate anti-tumor effects by boosting the immune response of T cells (14). Systemic therapies, including ICIs, have significantly improved the OS rate and quality of life of patients with HCC. However, the tumor microenvironment and the immune escape mechanism confer HCC with primary or adaptive resistance to these systemic therapies (14, 15). Only 10%–35% of patients experience sustained clinical benefits from these therapies (16). Currently, the 5-year survival rate of patients with advanced and intermediate HCC is less than 20% (11). Therefore, targeting the immune microenvironment of HCC, especially the activation and inhibition of T cells in this microenvironment, can significantly impact HCC therapy response and improve the prognosis of patients.

T cell proliferation regulators (TCRs), which are a diverse group of molecules, including proteins, enzymes, receptors, and signaling molecules, are involved in T-cell development, differentiation, proliferation, and function (17). In the tumor immune environment, the Expression and function of TCRs may be dysregulated, which can impair the ability of T cells to recognize and clear tumor cells. For example, CDK1 can influence tumor immunity by regulating the migration of immune cells into the bladder cancer microenvironment. CDK1 is also associated with tumor mutational burden (TMB) and microsatellite instability (18). CXCL12 is highly enriched in fibroblasts and can promote the proliferation of T cells in bladder cancer microenvironment. CXCL12 specifically interacts with CXCR4 expressed on the surface of T cells and macrophages (19). Cell cycle suppressor proteins, such as p27 and p21, play a major role in T-cell proliferation (20). The Expression of immune checkpoint molecules (such as PD-1 and CTLA-4) also affects the function of TCRs, which subsequently influence the anti-tumor activity of T cells (21). Therefore, targeting or modulating specific TCRs can potentially enhance the anti-tumor activity of T cells or improve the efficacy of immunotherapy. For example, inhibiting negative regulators in the tumor microenvironment or enhancing the Expression of positive regulators improves the cytotoxic effects of T cells against tumors. Additionally, TCRs serve as a marker for tumors to predict prognosis and guide treatment. In clear cell renal cell carcinoma (ccRCC), T-cell proliferation-related regulatory genes (TPRGs) are used to predict prognosis, identify tumor types (such as hot and cold tumors), and guide treatment (22). Previously, we analyzed the expression characteristics of 35 TPRGs and their somatic mutations in TPRG-associated subtypes, OS, biological pathways, and immunity in lung adenocarcinoma (LUAD) and developed a TPRG-associated risk model based on six genes. This model could accurately predict TPRG prognosis and immunotherapy response (23). However, the specific mechanism of TCRs in the tumor microenvironment and their roles in the immunotherapy response and prognosis of HCC have not been elucidated.

This study examined the role of TCRs in HCC using The Cancer Genome Atlas-Liver Hepatocellular Carcinoma (TCGA-LIHC) and International Cancer Genome Consortium-Liver Cancer-Riken, Japan (ICGC-LIRI-JP) datasets. The intersection of TCRs and differentially expressed genes (DEGs) between HCC and non-cancerous samples provided differentially expressed TCRs (DE-TCRs). Prognostic genes were identified using Cox regression analysis to establish an HCC risk model. The roles of these prognostic genes in HCC and their impact on prognosis were analyzed. Additionally, the mutational spectrum, immune cell infiltration, immunotherapy efficacy, and chemotherapy efficacy were examined. This study aimed to elucidate the effects of TCRs on the progression and prognosis of HCC and offer novel insights into HCC treatment strategies.

## 2 Materials and methods

### 2.1 Origins of the data

The RNA-sequencing data (HTSeq-Counts and HTSeq-FPKM) and clinical characteristics of the TCGA-LIHC cohort were extracted from the UCSC Xena database (http://xena.ucsc.edu/). TCGA-LIHC dataset, comprising the data of 50 non-cancerous and 365 HCC samples, served as the training set. The RNA-sequencing data and clinical information of the ICGC-LIRI-JP cohort were obtained from the ICGC database. The ICGC-LIRI-JP dataset, comprising the survival and gene expression data of 232 patients with HCC, served as the testing set. This study retrieved 35 TCRs from previous studies (19, 24). The immunophenoscore (IPS) and tumor immune dysfunction and exclusion (TIDE) scores of patients with HCC were obtained from The Cancer Imaging Archive and TIDE databases, respectively.

### 2.2 Differential expression and enrichment analyses

DEGs between non-cancerous and HCC samples in TCGA-LIHC datasets were identified using the R package DEseq2 (p < 0.05 and |log_2_ fold-change (FC)| > 0.5) (25). These DEGs were then overlapped with TCRs using the R package ggvenn (26) to obtain differentially expressed TCRs (DE-TCRs). The DE-TCRs were subjected to Gene Ontology (GO) and Kyoto Encyclopedia of Genes and Genomes (KEGG) using the clusterProfiler package with a significance threshold set at p < 0.05 (27).

### 2.3 Establishment of risk model

A risk model for HCC was developed using TCGA-LIHC and ICGC-LIRI-JP datasets. TCGA-LIHC dataset was subjected to univariate Cox regression analysis to identify DE-TCRs using the coxph function in the R package “survival” (27). Prognosis-related genes were screened based on the following conditions: hazard ratio (HR) ≠ 1, p < 0.05. Subsequently, the least absolute shrinkage and selection operator (LASSO) algorithm was employed to identify prognostic genes (family = “cox,” nfold = 10) (28). A risk model was established using TCGA-LIHC dataset. The risk score was calculated as follows: $risk\;score=\sum_{i=1}^{n}\left(Coef\left(i\right)\times expr\left(i\right)\right)$ . The HCC samples were divided into high-risk and low-risk groups based on the median risk score, and Kaplan-Meier (K-M) survival analysis was performed to assess the difference in survival between the two groups. Additionally, to further verify the validity of the risk model, we plotted receiver operating characteristic (ROC) curve and calculated the area under the curve (AUC) of the model to assess its accuracy. Meanwhile, validation was performed with the ICGC-LIRI-JP dataset. Finally, the significance of differential prognostic gene expression levels between HCC and non-cancerous samples in the TCGA-LIHC dataset was analyzed using the Wilcoxon test.

### 2.4 Constructing and evaluating nomogram models

Univariate and multivariate Cox regression analyses and proportional hazards assumption were used to identify independent prognostic factors. The clinicopathological factors (N-stage, risk score, gender, T-stage, race, age, M-stage, and grade) of the HCC cohort (TCGA-LIHC) were systematically analyzed (29). A nomogram model was constructed using these independent prognostic factors with the R package rms (30). ROC, calibration, and decision curve analysis (DCA) curves were generated to further verify the validity of the model.

### 2.5 Enrichment analysis

To investigate the biological pathways of prognostic genes, the KEGG background gene set (c2.cp.kegg.v2023.1.Hs.symbols.gmt), which was extracted from the Molecular Signature Database (MSigDB), was subjected to gene set variation analysis (GSVA) (p < 0.05) (27). The background gene set h.all.v2023.1.Hs.symbols.gmt was downloaded from the MSigDB to examine differential regulatory pathways between the high-risk and low-risk groups in TCGA-LIHC cohort (27). All genes in the risk groups were subjected to GSVA using the R package GSVA (31) and differential analysis using the R package limma (32).

### 2.6 Immunological analysis of HCC

To evaluate the association between the risk score and immune cell infiltration in patients with HCC, six types of immune cells were analyzed using the Tumor Immune Estimation Resource algorithm in the R package IOBR (33). To further assess differences in immune status among patients with HCC, immune-related pathways, and immune cell infiltration were analyzed using the single-sample gene set enrichment analysis (ssGSEA) algorithm within the R package ‘GSVA’ (significance threshold: p < 0.05) (31). The correlations between the risk score and immune cells, as well as between prognostic genes and differential immune checkpoints, were determined using Spearman’s rank correlation analysis.

### 2.7 Immunotherapy effect prediction and drug sensitivity analysis

Patient response to immunotherapy was assessed using the IPS and TIDE scores. Immunotherapy responses in the high-risk and low-risk groups in the TCGA-LIHC dataset were evaluated based on outcomes predicted by the TIDE algorithm. Additionally, the half-maximal inhibitory concentration (IC50) values of 138 drugs against HCC were evaluated using the R package pRRophetic (34) to determine the sensitivity of patients with HCC to these drugs.

### 2.8 Identification and analysis of molecular subtypes based on prognostic genes

To identify molecular subtypes based on prognostic genes in HCC samples from TCGA-LIHC dataset, consensus clustering was performed using the R package ‘ConsensusClusterPlus’ (35). The K-M curve was plotted using the R package survminer (36) to evaluate the differential OS between patients with different subtypes (p < 0.05). Additionally, differential immune cell infiltration statuses between these subtypes were analyzed (p < 0.05).

### 2.9 Mutation and copy number variation analyses

The data on the single nucleotide polymorphism mutation site of patients with HCC were extracted from the TCGA database. The R package maftools was used to analyze the mutation data of high-risk, low-risk, and prognostic genes (37). To elucidate the relationship between TMB and prognostic genes, the TMB of patients with HCC was calculated using the R package survminer (36). Based on the median risk score and the median TMB, patients with HCC in TCGA-LIHC dataset were grouped as follows to examine survival differences: H_TMB-H_risk, H_TMB-L_risk, L_TMB-H_risk, and L_TMB-L_risk. Additionally, somatic gene copy number data of TCGA-LIHC dataset were downloaded from the UCSC database (https://genome.ucsc.edu/). The R package OmicCircos (27) was used to analyze the chromosomal location of CNV in prognostic genes.

### 2.10 Molecular regulatory network construction

miRNAs targeting prognostic genes were predicted using the miRDB database (https://mirdb.org/). Subsequently, microRNAs (miRNAs) targeting long non-coding RNAs (lncRNAs) were predicted using the miRNet database (https://www.mirnet.ca/) to establish the mRNA-miRNA-lncRNA regulatory network. Transcription factors (TFs) regulating the prognostic genes were predicted using the Transcriptional Regulatory Relationships Unraveled by Sentence-based Text mining database. The mRNA-miRNA-lncRNA and miRNA/TF-gene regulatory networks were visualized using Cytoscape software.

### 2.11 Quantitative real-time polymerase chain reaction

Five pairs of non-cancerous and HCC samples were collected from the General Hospital of Ningxia Medical University. Informed consent was obtained from all participants. This study was approved by the Ethics Committee of the General Hospital of Ningxia Medical University (approval number: KYLL-2021-229).

Total RNA was extracted using the TRIzol reagent (Invitrogen, USA), following the manufacturer’s instructions. The isolated RNA was reverse-transcribed into complementary DNA (cDNA) using the SureScript First-strand cDNA Synthesis kit (Servicebio, China). The qRT-PCR analysis was performed with CFX Connect Thermal Cycler (Bio-Rad, USA). The relative expression levels of mRNAs were calculated using the 2^−ΔΔCt^ method. The sequences of all primers are shown in **Supplementary Table 1**.

### 2.12 Statistical analysis

Bioinformatics analyses were performed using R language (v. 4.2.2). The relationship between two variables was assessed using Spearman correlation analysis. Cox regression analysis was performed to determine the factors influencing the survival status. LASSO regression analysis was performed to reduce model complexity and avoid overfitting. K-M curves were plotted to determine the differential survival between the high-risk and low-risk groups. The significant differences between the survival curves were determined using the Log-rank test. ROC curves were plotted to assess the validity of the risk model. Data from different groups were compared using the Wilcoxon test (P < 0.05).

## 3 Results

### 3.1 Identification and functional enrichment analysis of DE-TCRs

**Figure 1:**
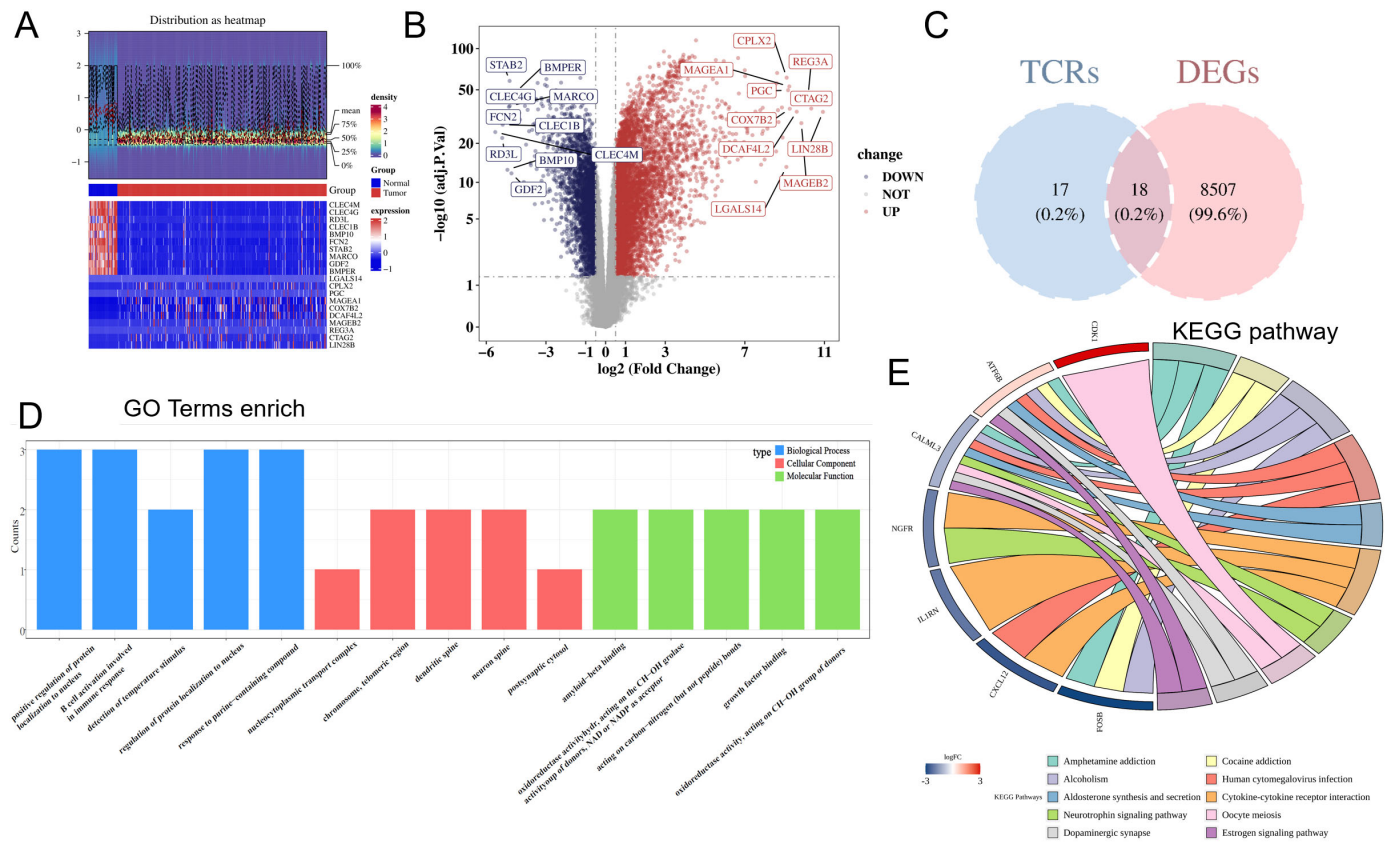
Identification and functional enrichment analysis of differentially expressed T-cell proliferation regulators (DE-TCRs). **(A)** The heatmap of differentially expressed genes. In the middle annotation bar, non-cancerous and HCC samples are indicated in blue and red colors, respectively. The intensity of the color in the heatmap signifies gene expression density per sample, with darker colors indicating higher density. The y-axis of the lower heatmap represents genes (red and blue colors indicate upregulation and downregulation, respectively). **(B)** Volcano plot of differentially expressed genes. The x-axis shows the log_2_ fold-change (FC) values, while the y-axis shows the −log_10_ (adjusted p-values). Each point represents a gene. The horizontal reference line represents −log_10_ (0.05) = 1.3, while the vertical reference line represents log_2_ (FC) = ± 0.5. Divided by the reference line, upper right genes are upregulated genes (red), and upper left genes are downregulated genes (blue). Gray represents genes with non-significant differences in expression levels. The top 10 upregulated genes and the top 10 downregulated genes with the largest log_2_ (FC) values are marked. **(C)** Venn diagram for DE-TCR identification. Pink and blue represent genes unique to the differentially expressed gene set and the TCR set, respectively. **(D)** Gene Ontology (GO) enrichment (Top 5). Each column represents a GO term. The color of the column represents different GO categories. The length represents the number of genes enriched in the term. **(E)** Kyoto Encyclopedia of Genes and Genomes (KEGG) enrichment. The color transition from red (high) to blue (low) indicates log_2_ (FC) values of the genes. Each pathway is represented by a distinct color.

In total, 8,525 DEGs were identified between HCC and non-cancerous samples (5,853 upregulated genes and 2,672 downregulated genes) (**Figures 1A, B**). The DEGs were intersected with TCRs to obtain 18 DE-TCRs (**Figure 1C**). DE-TCRs were enriched in various GO terms, including telomeric region, chromosome, positive regulation of protein localization to the nucleus, and amyloid-beta binding (**Figure 1D**). Additionally, DE-TCRs were enriched in several KEGG pathways, such as amphetamine addiction, alcoholism, and cytokine-cytokine receptor interaction (**Figure 1E**).

### 3.2 Prognostic genes screening and analysis

**Figure 2:**
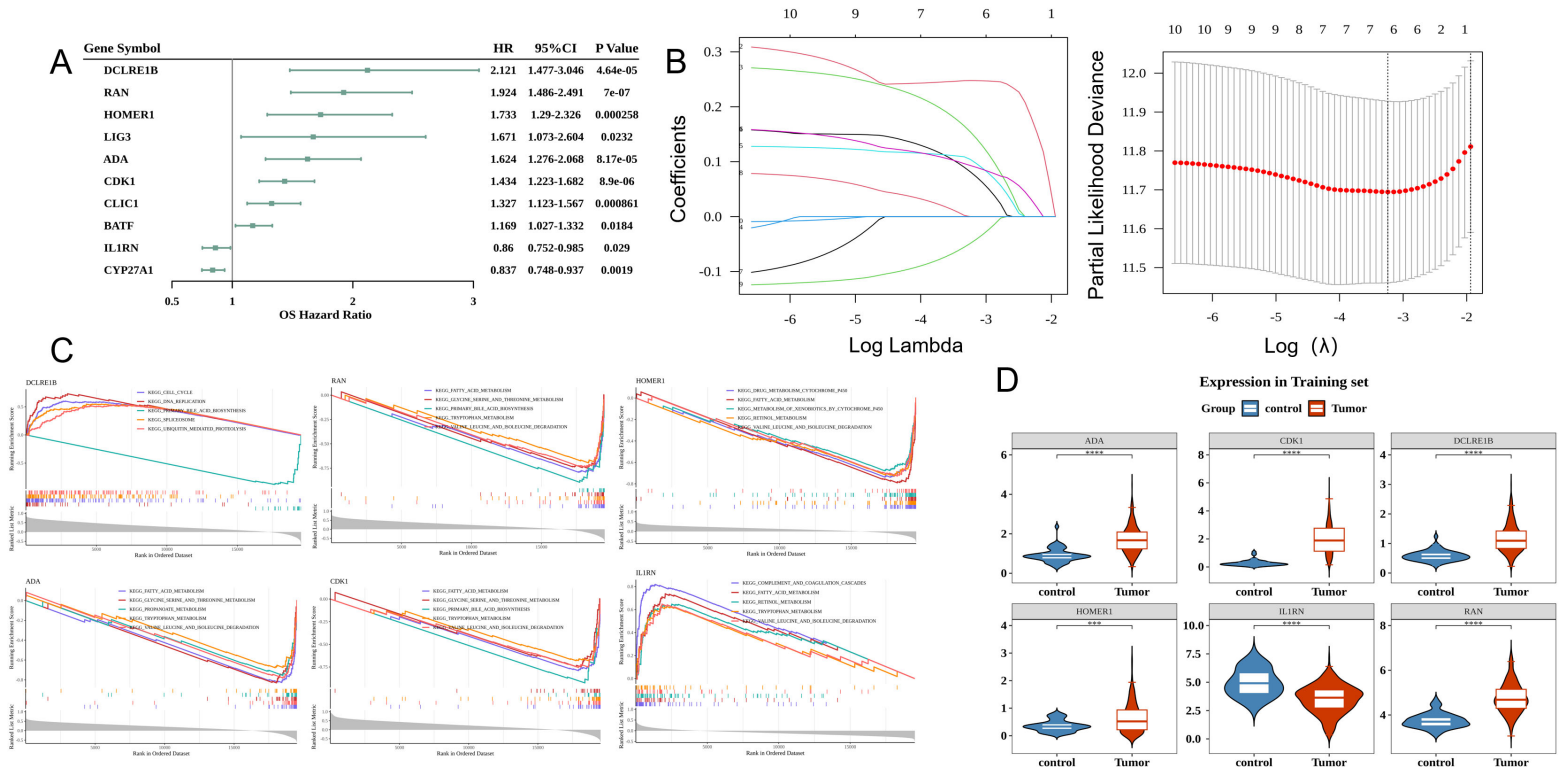
Prognostic gene screening and analysis. **(A)** Forest plot of Cox univariate analysis results. The leftmost side lists the prognostic genes. The three columns of numbers on the right represent the hazard ratio (HR) values corresponding to the gene, the 95% confidence interval of the HR value, and the p-value. **(B)** Least absolute shrinkage and selection operator (LASSO) analysis. The left figure demonstrates cross-validation with the middle line marking the minimum error. The optimal log (lambda) value is determined. Key genes and their coefficients are then identified in the right figure based on the lambda value. **(C)** Gene set enrichment analysis (GSEA) (Top 5) of characteristic genes. The figure has three sections. The top section displays the enrichment score calculated for each gene, linked into a line graph. The middle section visualizes the rank of each gene within the set. The bottom section depicts the overall rank distribution of all genes. **(D)** Analysis of the Expression of characteristic genes in hepatocellular carcinoma (HCC) versus control samples.

Univariate Cox regression analysis of 18 DE-TCRs revealed the following 10 prognosis-related genes (HR ≠ 1 and p < 0.05): *DCLRE1B*, *RAN*, *HOMER1*, *ADA*, *CDK1*, *IL1RN*, *CLIC1*, *BATF*, *LIG3*, and *CYP27A1* (**Figure 2A**). LASSO Cox regression analysis identified the following six prognostic genes: *DCLRE1B*, *RAN*, *HOMER1*, *ADA*, *CDK1*, and *IL1RN* (**Figure 2B**). GSEA revealed that the prognostic genes were significantly enriched in pathways related to primary bile acid biosynthesis (**Figure 2C**). All prognostic genes, except *IL1RN*, were upregulated in HCC (**Figure 2D**).

### 3.3 Prognostic gene-based risk model can predict HCC prognosis

**Figure 3:**
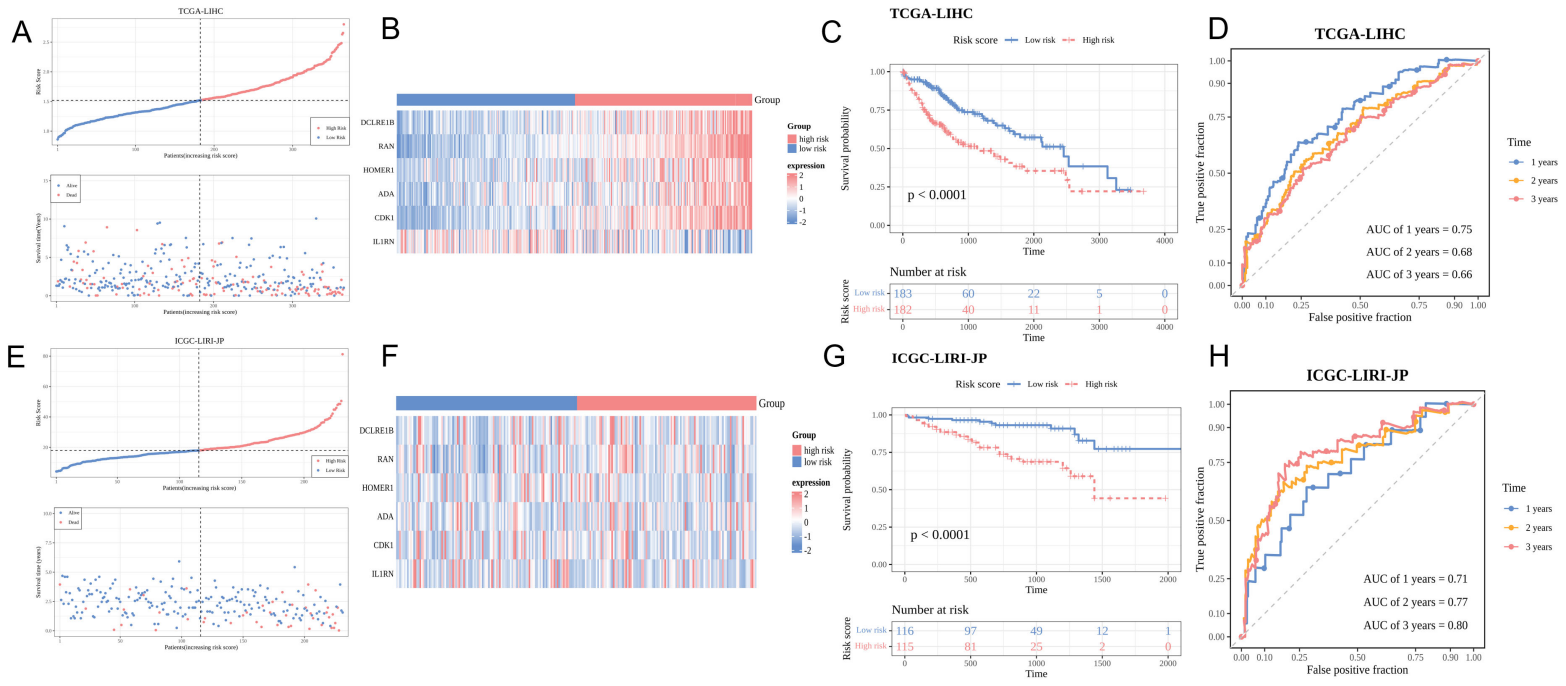
Predictive performance of the risk model. **(A, E)** Risk curve and survival status distribution of hepatocellular carcinoma (HCC) samples in the training and validation sets. The x-axis represents the risk score, increasing from left to right. In the upper part, red and blue points indicate high-risk and low-risk patients, respectively. In the lower part, red and blue dots denote deceased and surviving patients, respectively. **(B, F)** Characteristic gene expression analysis in the training and validation sets. The y-axis lists six characteristic genes. Red and blue colors indicate upregulation and downregulation, respectively. High-risk and low-risk groups are shown in red and blue colors, respectively. **(C, G)** Kaplan-Meier survival curves of patients in the training and validation sets. Red and blue represent high-risk and low-risk groups, respectively. **(D, H)** Receiver operating characteristic (ROC) curve of the training and validation sets.

**Figure 3A** shows the distribution of HCC samples in the two risk groups in TCGA-LIHC cohort. *DCLRE1B*, *RAN*, *HOMER1*, *ADA*, and *CDK1* were upregulated, whereas *IL1RN* was downregulated in the high-risk group (**Figure 3B**). K-M survival curve analysis indicated that patients in the high-risk group exhibited decreased survival probabilities (**Figure 3C**). Time-dependent ROC validation confirmed the efficacy of the model (area under the curve (AUC) values for predicting 1-year, 2-year, and 3-year survival were 0.75, 0.68, and 0.66, respectively) (**Figure 3D**). The findings of the ICGC-LIRI-JP dataset analysis were consistent with those of TCGA-LIHC dataset analysis (**Figures 3E–G**). The AUC values for predicting 1-year, 2-year, and 3-year survival in the ICGC-LIRI-JP dataset were 0.71, 0.77, and 0.80, respectively (**Figure 3H**).

### 3.4 Application of the nomogram model for HCC diagnosis

**Figure 4:**
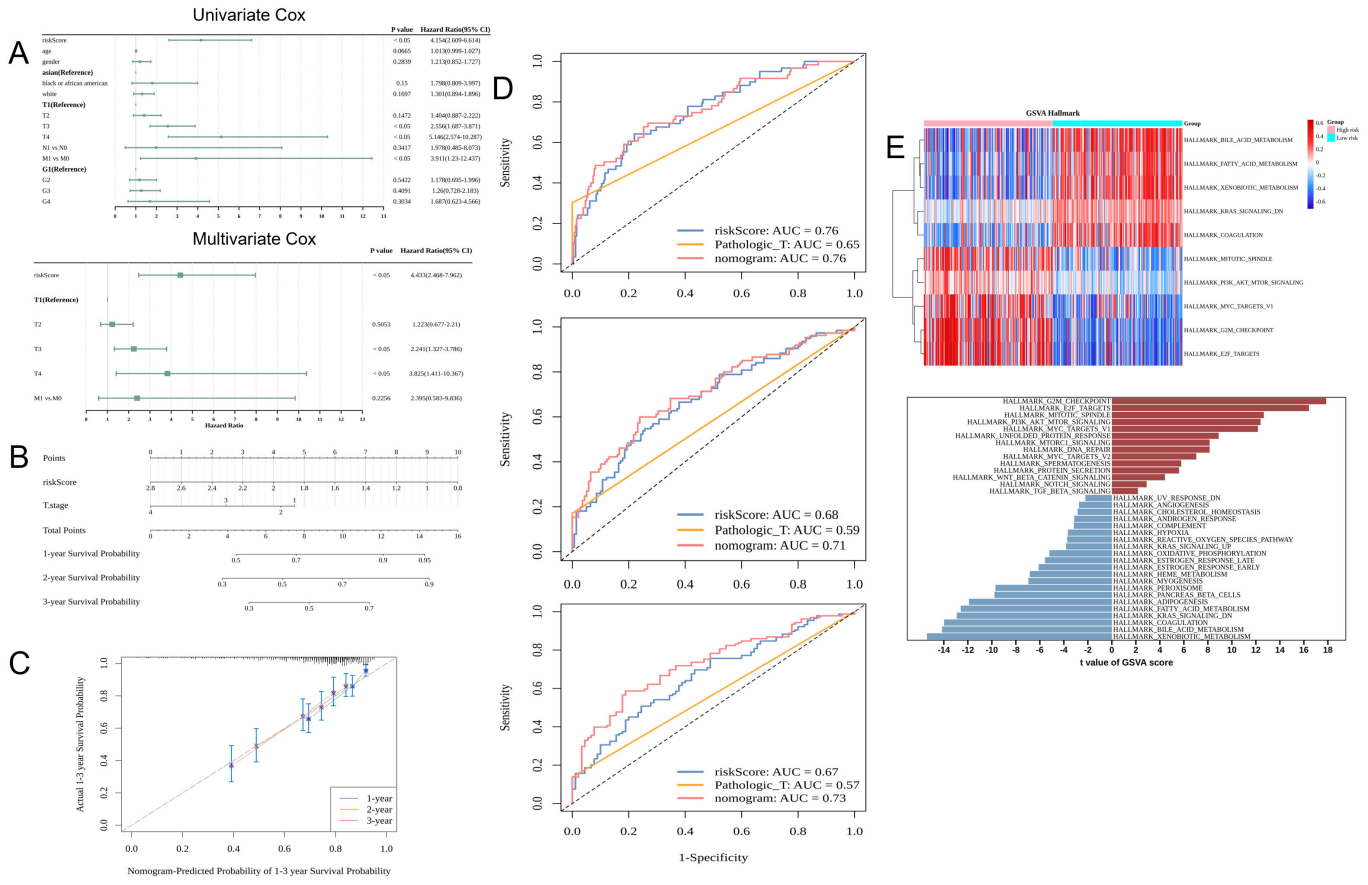
Application of the nomogram model for clinical hepatocellular carcinoma (HCC) cases. **(A)** Results of univariate and multivariate Cox regression analysis: The leftmost column shows the risk score and clinical characteristics, while the two right columns show the corresponding p-values and hazard ratio (HR) values. **(B)** Nomogram. **(C)** Nomogram calibration curves for predicting 1-year, 2-year, and 3-year survival rates. The x-axis shows the predicted event rate, while the y-axis shows the observed event rate (both ranging from 0 to 1). **(D)** The receiver operating characteristic (ROC) curves for predicting the 1-year, 2-year, and 3-year survival rates. **(E)** The upper panel shows the pathways activated and inhibited in the high-risk and low-risk groups (Top 5). The horizontal axis represents the samples (pink and blue colors indicate the high-risk and low-risk groups, respectively). The vertical axis represents the pathway (red and blue colors indicate the enriched and suppressed pathways, respectively). The lower panel shows the gene set variant analysis (GSVA) results.

Cox regression analysis identified the risk score and T-stage were independent prognostic factors (**Figure 4A**). A nomogram model was constructed based on these independent prognostic factors (**Figure 4B**). The calibration curves for 1-year, 2-year, and 3-year survival revealed that the predicted outcomes were consistent with the actual outcomes, indicating that the model can accurately predict HCC prognosis (**Figure 4C**). The AUC values of the nomogram for predicting 1-year, 2-year, and 3-year survival were more than 0.6. Thus, the predictive accuracy of the nomogram was higher than that of the risk score or T-stage alone (**Figure 4D**). In the high-risk group, 14 pathways were activated, including those related to immunization, such as HALLMARK_NOTCH_SIGNALING and HALLMARK_WNT_BETA_CATENIN_SIGNALING. In contrast, 21 pathways were inhibited in the high-risk group, including HALLMARK_COAGULATION and HALLMARK_ XENOBIOTIC_METABOLISM (**Figure 4E**).

### 3.5 Immune cell infiltration was upregulated in high-risk patients with HCC

**Figure 5:**
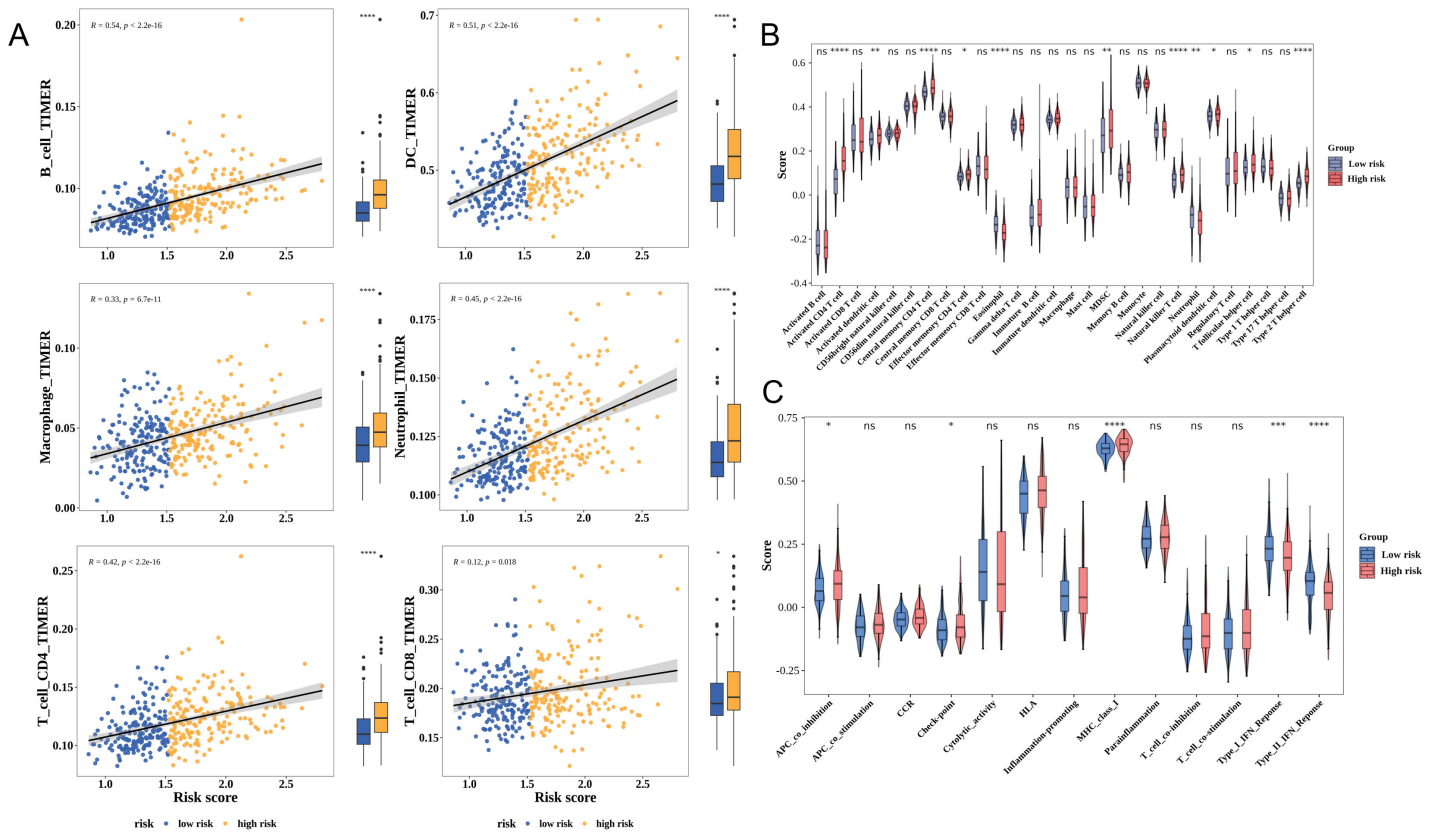
Correlation between risk scores and the tumor microenvironment of hepatocellular carcinoma (HCC). **(A)** The Tumor Immune Estimation Resource (TIMER) algorithm assesses the relationship between the risk score and immune cell infiltration. Yellow and blue represent the high-risk and low-risk groups, respectively. Each image is divided into two parts: correlation analysis (left) and differential analysis (right). **(B)** Analysis of differential immune cell infiltration levels using the single-sample gene set enrichment analysis (ssGSEA) algorithm. **(C)** Analysis of differential immune-related pathways using the ssGSEA algorithm. * p< 0.05, ** p< 0.01, *** p< 0.001, **** p< 0.0001.

The development of tumors is closely linked to the immune microenvironment. Immune cell infiltration was positively correlated with the risk score in patients with HCC. The infiltration levels of six immune cell types were upregulated in the high-risk group (**Figure 5A**). Meanwhile, the infiltration levels of 11 immune cell types, including eosinophil, varied between the high-risk and low-risk groups. The infiltration levels of most of these 11 immune cell types were upregulated in the high-risk group (**Figure 5B**). Additionally, the levels of five immune-related pathways, including the type II IFN response, varied between the high-risk and low-risk groups (**Figure 5C**).

### 3.6 Prognostic genes affect the immunotherapeutic response of HCC

**Figure 6:**
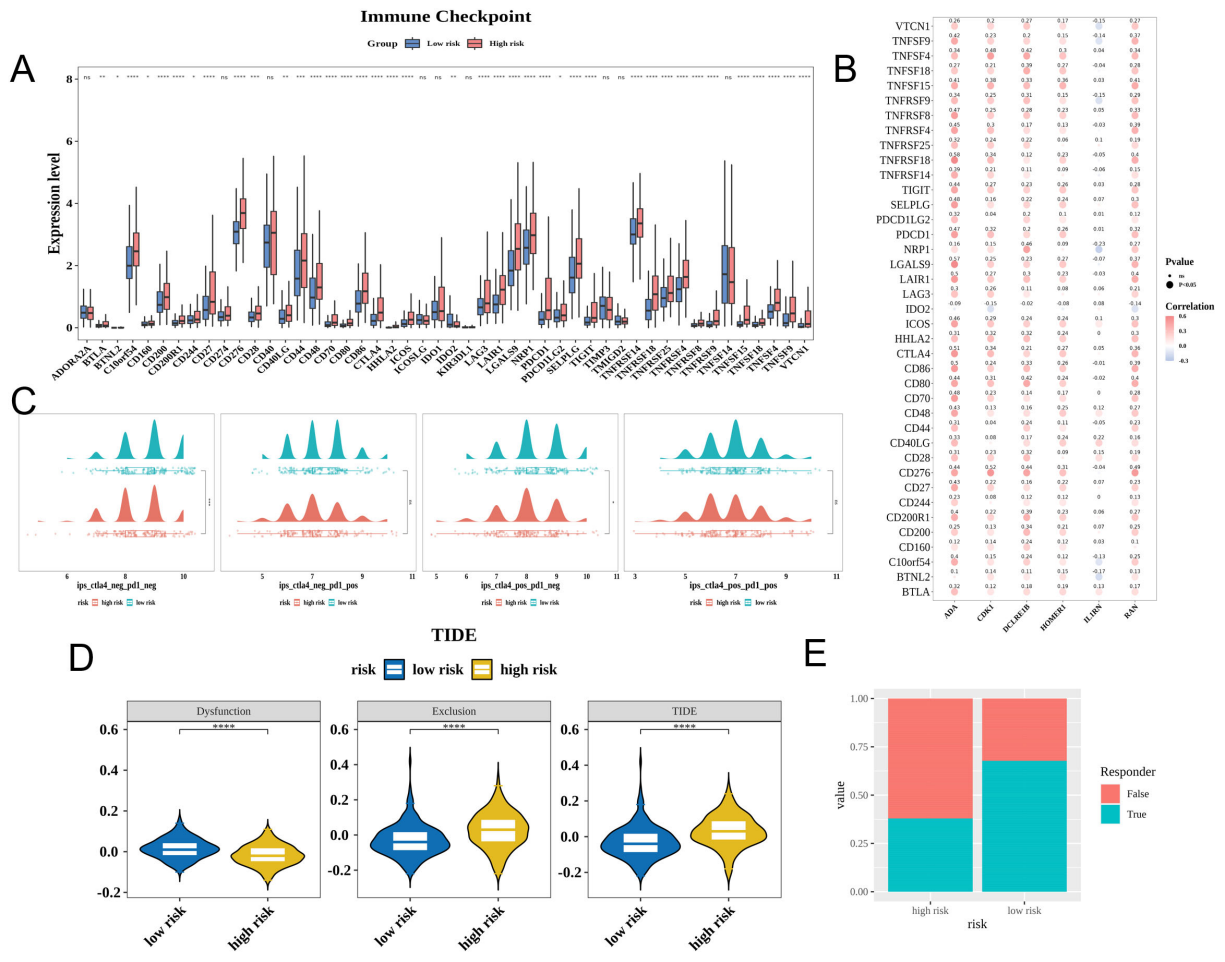
Prognostic genes can affect the immunotherapy efficacy in patients with hepatocellular carcinoma (HCC). **(A)** Expression of immune checkpoints in the high-risk and low-risk groups. **(B)** Correlation between characteristic genes and differential immune checkpoints (Dot size represents significance; red and blue colors indicate positive and negative correlations, respectively; darker shades indicate stronger correlations). **(C)** Immunephenoscore (IPS) of the high-risk and low-risk groups (red and green colors indicate high-risk and low-risk, respectively). Each image features a density plot at the top and a scatter plot with a box plot at the bottom. **(D)** Tumor immune dysfunction and exclusion (TIDE) scores of the high-risk and low-risk groups (yellow and blue colors indicate the high-risk and low-risk groups, respectively). **(E)** Immune response ratio of patients in the high-risk and low-risk groups. * p< 0.05, ** p< 0.01, *** p< 0.001, **** p< 0.0001.

Immune checkpoints play a crucial role in mitigating autoimmune effects. In TCGA-LIHC cohort, 39 immune checkpoints were differentially expressed between the high-risk and low-risk groups (**Figure 6A**). The expression levels of most prognostic genes were positively correlated with those of 39 immune checkpoints. *ADA* exhibited the strongest positive correlation with *TNFRSF18* (cor = 0.58), whereas *IL1RN* exhibited the strongest negative correlation with *NRP1* (cor = −0.23) (**Figure 6B**). The levels of CTLA4-negative response and PD-1-negative response (ips_ctla4_neg_pd1_neg) and CTLA4-positive and PD-1-negative response (ips_ctla4_pos_pd1_neg) were downregulated in the high-risk group (**Figure 6C**). Furthermore, the high-risk group exhibited increased exclusion scores and decreased dysfunction scores, indicating that immune escape in this group was predominantly due to immune exclusion, which contributes to decreased responses to immunotherapy (**Figures 6D, E**).

### 3.7 Differential survival rates of HCC subtypes classified according to prognostic genes

**Figure 7:**
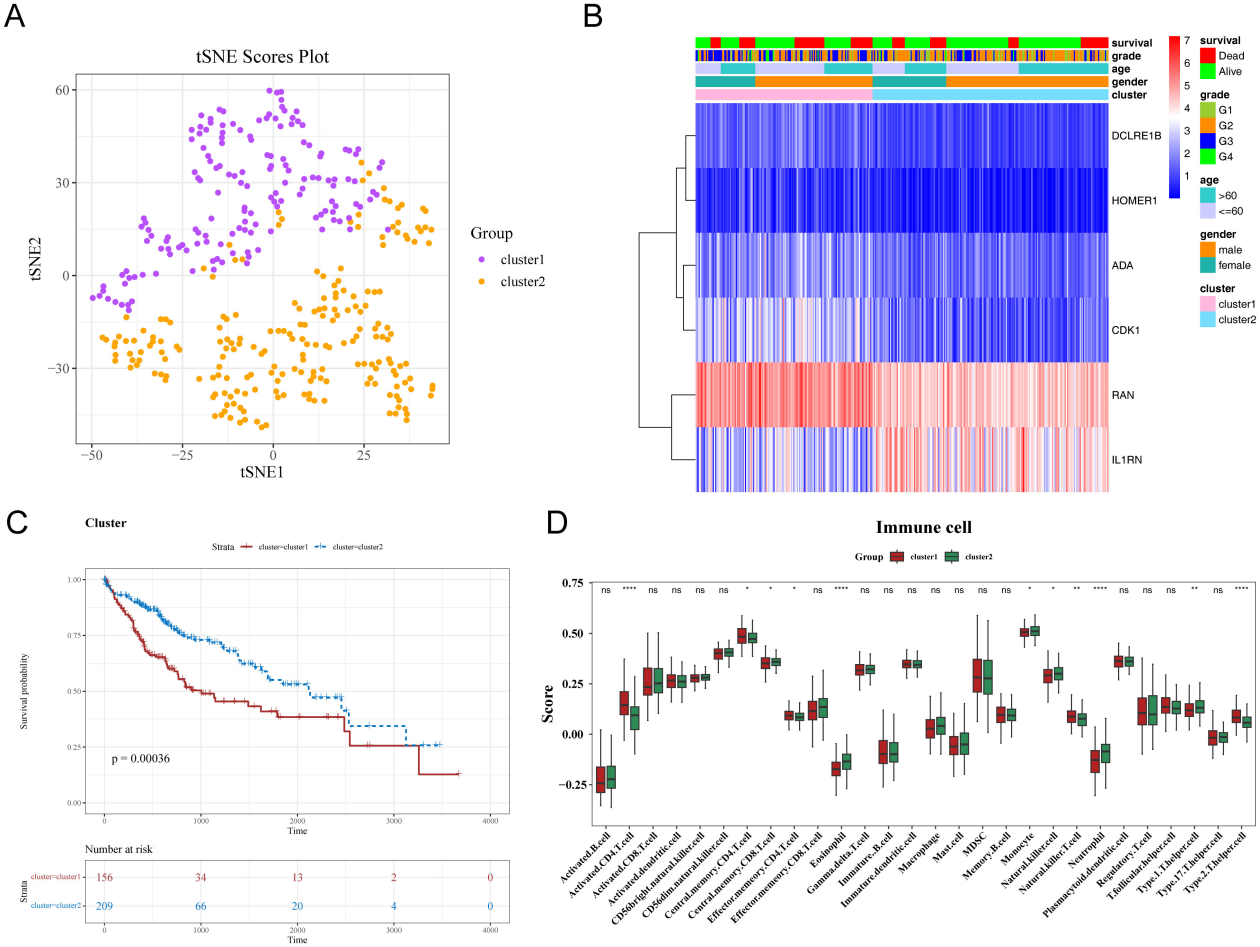
Differential characteristics of different subtypes of hepatocellular carcinoma (HCC). **(A, B)** Consistent clustering of characteristic genes in patients with HCC. **(C)** Kaplan-Meier (K-M) curves of HCC subtypes. The x-axis represents overall survival time in days, while the y-axis shows survival probability. Different colors represent different HCC subtypes. **(D)** Differential immune cell infiltration levels in HCC subtypes. * p< 0.05, ** p< 0.01, **** p< 0.0001.

HCC samples were categorized into the following two subtypes via consensus clustering: cluster 1, comprising 156 samples; and cluster 2, comprising 209 samples. The t-distributed stochastic neighbor embedding (tSNE) dimensionality reduction analysis distinguished the two subtypes, revealing variations in prognostic gene expression (**Figure 7A**). The K-M curve analysis revealed that the OS significantly varied between cluster 1 and cluster 2 with cluster 1 exhibiting poor survival (**Figures 7B, C**). Additionally, the infiltration levels of 11 immune cell types, including eosinophils, varied between cluster 1 and cluster 2 (**Figure 7D**).

### 3.8 Drug sensitivity and mutation profiles varied between two risk groups

**Figure 8:**
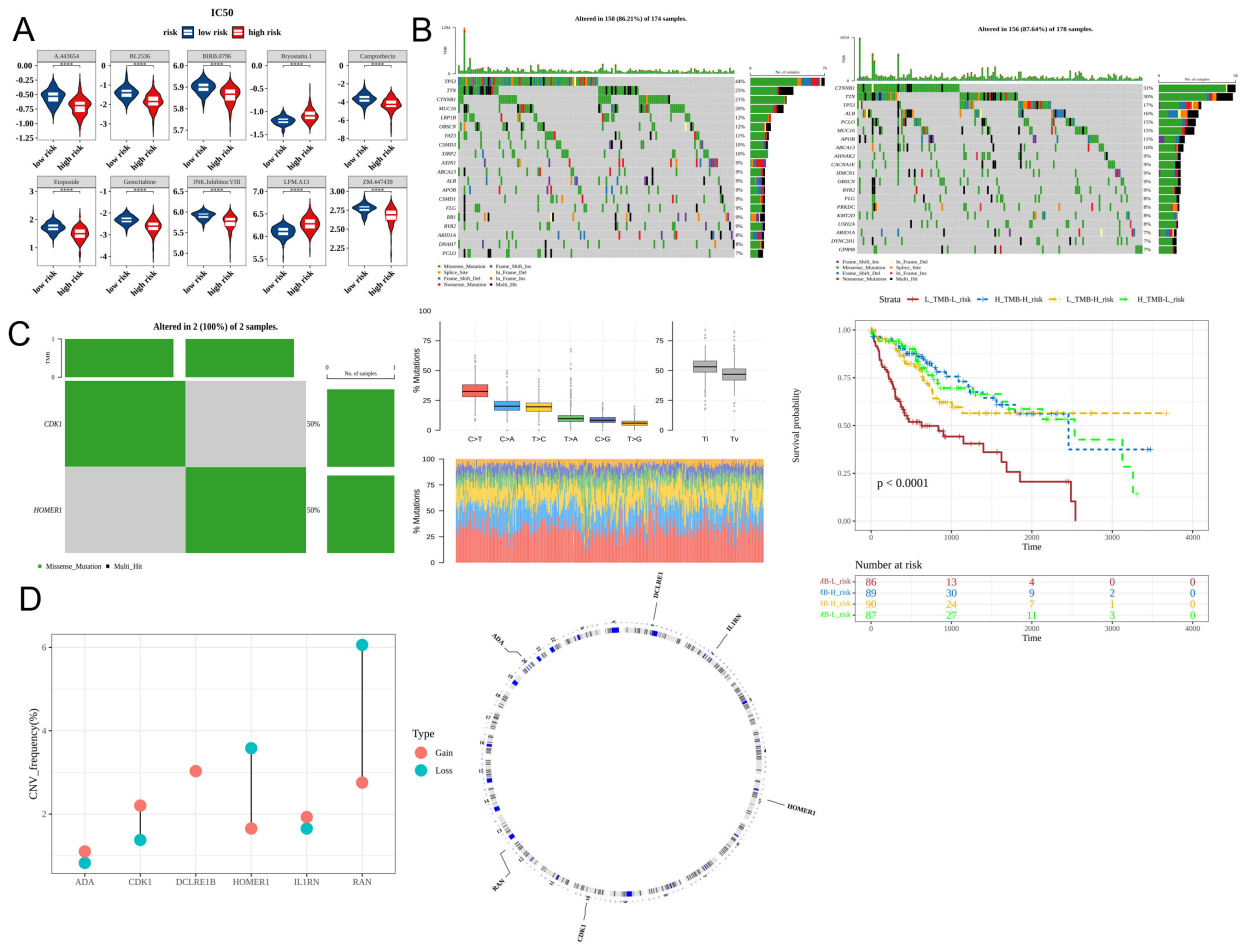
Differential drug sensitivity and mutation profiles between the high-risk and low-risk groups. **(A)** The half-maximal inhibitory concentration (IC50) values of the top 10 drugs in patients belonging to the high-risk and low-risk groups (Top 10). **(B)** Mutation profiles of the high-risk and low-risk groups (the top and bottom sections represent the low-risk and high-risk groups, respectively). **(C)** Analysis of characteristic gene mutations and Kaplan-Meier (K-M) curves based on tumor mutational burden (TMB) and risk score. **(D)** Copy number variation of characteristic genes (red and blue represent amplification and deletion, respectively). **** p< 0.0001.

Chemotherapy is a common treatment for malignant tumors. Analysis of 138 anti-cancer drugs revealed that the IC50 values of 108 drugs varied between the high-risk and low-risk groups. **Figure 8A** shows the 10 drugs (including A.443654, BI.2536, and BIRB.0796) with the most marked differences in IC50 values (**Figure 8A**). The frequency of mutations, predominantly missense mutations, in genes, such as *TP53*, *TTN*, and *CTNNB1*, varied between the high-risk and low-risk groups (**Figure 8B**). Additionally, the two prognostic genes *CDK1* and *HOMER1* also exhibited mutations. The frequency of transition (Ti) mutations was higher than that of transversion (Tv) and missense mutations (**Figure 8C**). CNV analysis revealed that the most frequent copy number increase was observed in *DCLRE1B*, whereas the most frequent copy number decrease was observed in *RAN* (**Figure 8D**).

### 3.9 Six prognostic genes were connected to several molecular regulatory systems

The lncRNA-miRNA-mRNA regulatory network comprised 350 nodes and 838 edges. The nodes included five prognostic genes (*RAN*, *HOMER1*, *IL1RN*, *DCLRE1B*, and *CDK1*), 21 miRNAs (such as hsa-miR-181a-5p and hsa-miR-122-5p), and 324 lncRNAs. This network featured competing endogenous RNA (ceRNA) interactions, such as DCLRE1B-hsa-miR-101-3p-XIST and IL1RN-hsa-miR-122-5p-ASB16-AS1 interactions (**Supplementary Figure 1A**). The miRNA/TF-prognostic gene regulatory network graph comprised 224 nodes and 227 edges. The nodes included three prognostic genes (*ADA*, *CDK1*, and *IL1RN*), 15 TFs (such as *SP1* and *RB1*), and 206 miRNAs. Examples of the miRNA/TF-gene interactions were *ADA*-*SP1* and *CDK1*-hsa-miR-374c-5p interactions (**Supplementary Figure 1B**).

### 3.10 HOMER1, ADA, and CDK1 were upregulated in patients with HCC

**Figure 9:**
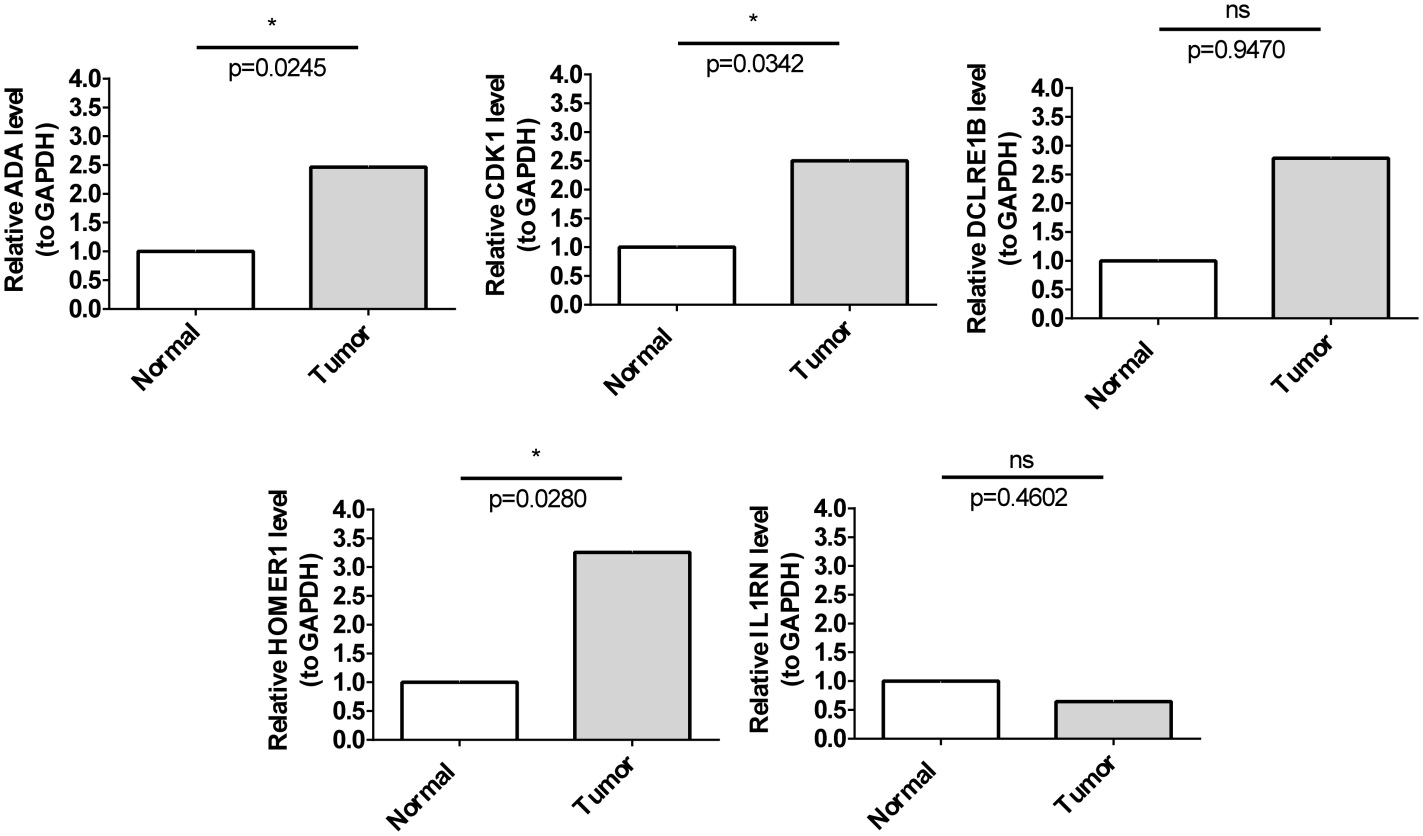
Validation of prognostic genes. The mRNA expression levels of five prognostic genes in cancer and para-cancerous tissues. * p< 0.05.

qRT-PCR analysis indicated that the mRNA expression levels of *HOMER1*, *ADA*, and *CDK1* in HCC samples were significantly higher than those in non-cancerous samples. However, the mRNA expression levels of *IL1RN* and *DCLRE1B* were not significantly different between HCC and non-cancerous samples (**Figure 9**). The expression patterns of *HOMER1*, *ADA*, and *CDK1* determined using qRT-PCR analysis were consistent with those determined using bioinformatic analysis.

## 4 Discussion

HCC is the predominant form of primary liver cancer, accounting for more than 90% of primary liver cancer cases. Globally, HCC is the sixth most common cancer and the third leading cause of cancer-related deaths (38). The poor prognosis of HCC is because patients with early-stage HCC do not exhibit prominent symptoms. Thus, HCC is mostly diagnosed at an advanced stage, which is associated with resistance to conventional chemotherapy and radiotherapy. The survival rates of patients with HCC are low due to the development of treatment resistance and disease recurrence (39). The emergence of cancer immunotherapy, which stimulates the immune system, especially T cells, to target and kill tumor cells, has revolutionized HCC treatment, offering new hope to patients with HCC (40, 41). The proliferation of T cells, which aggregate and cluster in the tumor microenvironment, serves as a marker of tumor reactivity and is positively correlated with the outcomes of ICI therapy (42–44). However, T-cell proliferation is not an infallible measure of tumor reactivity and outcomes (45–50). Recently, Legut et al., who were the first to identify TPRGs (24), identified several TPRGs that enhance T-cell function. The understanding of the role of TCRs in HCC immunotherapy response can aid in guiding the selection of immunotherapy regimens for patients with HCC.

This study systematically investigated the Expression of TCRs and their relationship with OS in HCC. In total, 8,525 DEGs were identified between HCC and non-cancerous samples (5,853 upregulated genes and 2,672 downregulated genes) in TCGA-LIHC dataset. Based on the filtering criteria for TCRs, 18 DE-TCRs were selected. Next, a novel prognostic model comprising six prognostic genes (*DCLRE1B*, *RAN*, *HOMER1*, *ADA*, *CDK1*, and *IL1RN*) was established using LASSO regression analysis.

DCLRE1B, a 5’-3’ exonuclease belonging to the metallo-β-lactamase superfamily, is one of the evolutionarily conserved genes and is involved in DNA interstrand cross-linking (ICL) repair (51). DNA ICL induces tumor cell death (52). Lee et al. reported that the *Oldenlandia diffusa* extract promotes cell death in cisplatin-resistant ovarian cancer cells by regulating DCLRE1B. Additionally, the upregulated Expression of *DCLRE1B* in the liver, kidney, and pancreatic cancers is associated with poor prognosis (53). Li et al. reported that DCLRE1B expression was significantly correlated with immune cells in various cancers. The authors reported that DCLRE1B expression was associated with immune checkpoint gene expression and immune therapy sensitivity (54). *In vitro* experiments demonstrated that DCLRE1B facilitates the proliferation and migration of pancreatic cancer cells. The upregulated Expression of DCLRE1B in human cancers promotes cancer initiation and progression by modulating processes, such as immune cell infiltration (54). Recent studies have confirmed the role of DCLRE1B in immune regulation and tumor immunotherapy response (53). In this study, DCLRE1B was upregulated in HCC and regulated HCC occurrence and progression by modulating T cells, which was consistent with the findings of recent studies.

HOMER1, a member of the Homer family of dendritic proteins, is a scaffold protein that regulates glutamatergic synapses and spine morphogenesis (55). In this study, HOMER1 was upregulated in HCC and was negatively correlated with OS. This finding was consistent with that of Yang et al. (23), who demonstrated that *HOMER1* is a T-cell proliferation-related regulatory gene in LUAD. Experimental validation in LUAD cells revealed that HOMER1 can inhibit tumor cell proliferation, migration, and invasion (23).

RAN, a small GTP-binding protein of the RAS superfamily, is crucial for protein transport through the nuclear pore complex (56). Elsalahaty et al. revealed that the RAN*rs14035 variant may be an independent risk factor for HCC and that RAN is involved in miRNA synthesis and promoting the development of various cancers, including HCC (57). In this study, *RAN* was upregulated in HCC and regulated tumor development and progression by influencing T lymphocyte responses in the tumor microenvironment. CDK1, a serine/threonine protein kinase, plays a crucial role in the G1/S and G2/M phase transitions of the cell cycle (58, 59). The upregulated Expression of CDK1 in HCC is associated with poor prognosis. The downregulation or the inhibition of CDK1 overexpression can improve survival outcomes (60–62). Chen et al. reported that IRF-1 can regulate the transcription of CDK1, playing a crucial role in both pathological and physiological phenomena, such as viral infection, carcinogenesis, pro-inflammatory damage, and immune system development (63). In this study, *CDK1* was upregulated in HCC, serving as a risk gene. *CDK1* was upregulated in patients with high-risk scores and was significantly associated with poor prognosis.

ADA, which is critical for purine nucleoside and DNA metabolism, plays key roles in the immune and vascular systems (64). The activity of ADA is reported to be upregulated in patients with cancer. The upregulated ADA activity is associated with the staging of gastric, bladder, breast, colorectal, and renal cancers (65–73). In liver cancer, elevated ADA is correlated with serum atezolizumab concentrations and impaired functions of CD8-positive T cells, including suppressed secretion of interferon-γ and tumor necrosis factor-α. This suggests that high ADA levels may decrease atezolizumab exposure, potentially reducing its anti-cancer efficacy (74). Immune cell death and DNA damage response play significant roles in cancer progression and prognosis (75). Zhang et al. identified *ADA* as a prognostic gene associated with immune cell death and DNA damage response and that *ADA* is upregulated in HCC and contributes to the anti-tumor immune response in liver cancer (76). IL1RN, which serves as a natural interleukin-1 receptor antagonist (77), is negatively correlated with the proliferation of bladder cancer cells (78). Zhang et al. demonstrated that the upregulation of IL1RN-201/203 and anakinra treatment in KRAS-mutant intrahepatic cholangiocarcinoma mice significantly enhanced the anti-tumor immune response by altering neutrophil recruitment and phenotype. The upregulation of IL1RN-201/203 levels was associated with a favorable response to anti-PD-1 immunotherapy (79). Additionally, one study examining genes related to metabolic risk factors in non-alcoholic fatty liver disease and HCC reported that *IL1RN* is a protective prognostic gene for HCC. However, the biological function of IL1RN in HCC has not been elucidated (80, 81). In this study, *IL1RN* was downregulated in HCC, indicating its role as a protective gene. The Expression of *IL1RN* was downregulated in high-risk patients. These results are consistent with those of previous studies.

These findings indicated that TCRs play crucial roles in various processes, including T-cell proliferation and tumor immunotherapy response, highlighting the validity of the prognosis model developed in this study. To further validate the bioinformatics analysis results, the mRNA levels of five genes were examined in HCC tissues and adjacent tissues. The expression trends of *HOMER1*, *ADA*, and *CDK1* were consistent between qRT-PCR and bioinformatics analyses. However, the *IL1RN* and *DCLRE1B* mRNA levels were not significantly different between HCC and non-cancerous samples, which can be attributed to sample heterogeneity. Thus, the experimental results were consistent with the bioinformatics analysis results, indicating the reliability of the model.

GSVA revealed that the DNA replication and repair pathways were significantly enriched in high-risk patients, suggesting increased genomic alterations. Genomic mutation analysis revealed an increased frequency of mutations, including *TP53*, *TTN*, and *CTNNB1* mutations, in high-risk cases, indicating increased genomic instability. Somatic mutations in *TP53*, which are the most common alterations in human cancers, are associated with poor prognosis (82, 83). The increased frequency of these mutations in the high-risk group suggests decreased survival duration. Previous studies have suggested that *TP53* mutations affect the progression and prognosis of HCC and are associated with the immune microenvironment of HCC (84, 85). Therefore, *CTNNB1* mutations can serve as biomarkers for evaluating the effectiveness of immunotherapy in HCC (86–88). Huo et al. demonstrated that *CTNNB1* mutations are associated with poor prognosis and decreased disease-free survival (89). Consistently, this study demonstrated that the rate of *CTNNB1* mutations was upregulated in the high-risk group and that the increased rate of *CTNNB1* mutations was associated with decreased OS. Additionally, one study reported that *TP53* and *TTN* exhibited the highest mutation rates and that these mutations served as cancer-driving factors in hepatitis B virus-related HCC (90). These findings suggest that patients in the high-risk group are sensitive to DNA-damaging agent-based therapies due to enhanced genomic instability.

The tumor microenvironment exerts regulatory effects on tumor phenotypes. Immune cell infiltration, which is crucial for the immune evasion of tumor cells and the induction of inflammation, is a key feature of the tumor environment (91). This study examined the differential immune cell infiltration levels between the high-risk and low-risk groups. The risk scores were positively correlated with the presence of B cells, CD4+ T cells, CD8+ T cells, macrophages, neutrophils, and dendritic cells. Previous studies have demonstrated that CD4+ and CD8+ T cells suppress HCC development through the induction of anti-tumor immune responses (92). The proportion and quantity of CD4+ T cells are reported to be significantly upregulated in the peritumoral area of HCC tissues, promoting HCC progression (93). The presence of CD8+ T cells is associated with prolonged OS (94). Recent studies have reported a close correlation between intra-tumoral dendritic cell infiltration and poor prognosis in patients with HCC (95), which is consistent with the findings of this study. The infiltration of neutrophils in HCC is linked to adverse clinical outcomes (96). Neutrophils, which are involved in the activation, regulation, and effector functions of immune cells (97), accelerate HCC progression by secreting various cytokines (100). Increased macrophage infiltration is associated with poor prognosis in HCC (98). Macrophage infiltration in the tumor microenvironment promotes tumor growth, angiogenesis, invasion, and metastasis (99). Targeting macrophages is a promising adjuvant immunotherapy approach for patients with HCC (100, 101). The enrichment of immune-related pathways varied between the high-risk and low-risk groups. The TIDE score in the low-risk group was lower than that in the high-risk group. This suggests that patients in the low-risk group can potentially benefit from immunotherapy. In high-risk patients with HCC, high levels of immune cell infiltration may not indicate that the immune system can effectively control or clear tumor cells. Immune cell functions are suppressed in the immunosuppressive microenvironment or the cancer cells evade immune system recognition and attack through multiple mechanisms, impairing the anti-tumor functions of immune cells. Previous studies have demonstrated that myeloid-derived suppressor cells exert immunosuppressive effects in HCC by expanding immune checkpoint signaling and suppressing the cytotoxic activity of natural killer cells. Additionally, cancer cells evade T-cell recognition due to the lack of a transporter protein or β2 macroglobulin associated with major histocompatibility complex class 1 antigen presentation (102, 103). Thus, this study contributed to the elucidation of unique mechanisms of the HCC immune microenvironment, which may aid in developing novel therapeutic strategies, including immunotherapeutic strategies, for HCC (104). The results of this study are consistent with those of previous studies on immune infiltration in HCC. This study offers valuable direction and guidance for understanding the mechanisms of immune cells in HCC. However, further studies are needed to elucidate specific mechanisms.

Increasing the sensitivity of HCC to various drugs can benefit patients, although further studies are needed to elucidate the specific underlying mechanisms (105). Analysis of drug sensitivity and risk scores revealed that the IC50 values of 5-fluorouracil, sorafenib, and VX-11e were low in the high-risk group, indicating their enhanced efficacy in this group. Conversely, the IC50 values of gefitinib, ceritinib, and sunitinib were high in the low-risk group, suggesting their decreased efficacy in this group (106). Thus, the prognostic model established in this study may facilitate the development of improved treatment strategies for HCC.

Six prognostic genes were used to construct a model to quantitatively assess the prognosis of patients with HCC. Patients with high-risk scores exhibited significantly decreased OS. The AUC values for predicting 1-year, 2-year, and 3-year OS were 0.75, 0.68, and 0.66, respectively. Univariate and multivariate analyses revealed that the risk scores and T-stage were independent prognostic factors. This indicated the relevance of the prognostic model for patients with late-stage HCC. Gene testing data and clinical characteristics should be integrated in the future for a comprehensive assessment. In this study, a nomogram combining risk scores and clinical factors was developed to predict the 1-year, 2-year, and 3-year OS of patients with HCC. The AUC values for predicting 1-year, 2-year, and 3-year were more than 0.7. Calibration curves revealed that the predicted survival rates concurred with the actual survival rates. The ROC curves demonstrated excellent predictive performance of the nomogram. Thus, the model developed in this study can quantitatively predict the prognosis of patients with HCC and enable the customization of treatment plans. This scoring system will aid healthcare professionals in predicting survival and selecting optimal treatment options.

This study has several limitations. The sample size was small in this study. Additionally, the data were obtained from public datasets. Furthermore, the accuracy and stability of the prognostic model were not validated through clinical studies. Finally, only the transcript levels were evaluated in this study, which may not reflect protein levels. Future studies must focus on overcoming these limitations.

In conclusion, this study established a novel prognostic model based on TCRs to predict the clinical outcomes of patients with HCC. Six TCR genes were correlated with HCC prognosis. The findings of this study may enable the development of novel diagnostic and therapeutic strategies for HCC.

## Data Availability

The raw data supporting the conclusions of this article will be made available by the authors, without undue reservation.

## Supplementary material

Supplementary Figure 1 Construction of competing endogenous RNA (ceRNA) and microRNA (miRNA)/transcription factor (TF)-gene networks. **(A)** ceRNA network of characteristic genes (red squares, blue diamonds, and yellow circles indicate characteristic genes, miRNAs, and long non-coding RNAs (lncRNAs) (degree ≥ 8), respectively). **(B)** miRNA/TF-gene network (red squares, purple diamonds, and yellow circles indicate characteristic genes, miRNAs (degree > 1), and TFs, respectively).
