# Supplementary material for: Prognostic modeling of hepatocellular carcinoma based on T-cell proliferation regulators: a bioinformatics approach
Source: Front Immunol. 2024 Oct 9;15:1444091. doi: 10.3389/fimmu.2024.1444091 (PMC11496079; doi:10.3389/fimmu.2024.1444091)
Supplement: Supplementary file 1 [file Table1.docx]

**Supplementary Table S1** qRT-PCR primer sequences

| **Primers** | **Sequences** | |
| --- | --- | --- |
| IL1RN F | AGACCTCCTGTCCTATGAGGC | |
| IL1RN R | TCGGCAGATCGTCTCCTTTG | |
| DCLRE1B F | TTGCTCTGCTGGGCTCTTTC |  |
| DCLRE1B R | CAGGCTCCAGAAGTCCACTG |  |
| HOMER1 F | GAAAGCAGCACCCTCTTCTC |  |
| HOMER1 R | CACCCCTACTCCTCGTCTCT |  |
| ADA F | GGAACCAGGCTGAACTGGTC |  |
| ADA R | TGGTCTTCCAGGGTGTGGTA |  |
| CDK1 F | CGTAGCTGGGCTCTGATTGG |  |
| CDK1 R | CAAACTCACCGCGCTAAAGG |  |
| internal reference-GAPDH F① | CGAAGGTGGAGTCAACGGATTT |  |
| internal reference-GAPDH R① | ATGGGTGGAATCATATTGGAAC |  |
| internal reference-GAPDH F② | CGAAGGTGGAGTCAACGGATTT |  |
| internal reference-GAPDH R② | ATGGGTGGAATCATATTGGAAC |  |
| internal reference-GAPDH F③ | CGAAGGTGGAGTCAACGGATTT |  |
| internal reference-GAPDH R③ | ATGGGTGGAATCATATTGGAAC |  |
