# Supplementary figures and images for: Prognostic modeling of hepatocellular carcinoma based on T-cell proliferation regulators: a bioinformatics approach
Source: Front Immunol. 2024 Oct 9;15:1444091. doi: 10.3389/fimmu.2024.1444091 (PMC11496079; doi:10.3389/fimmu.2024.1444091)

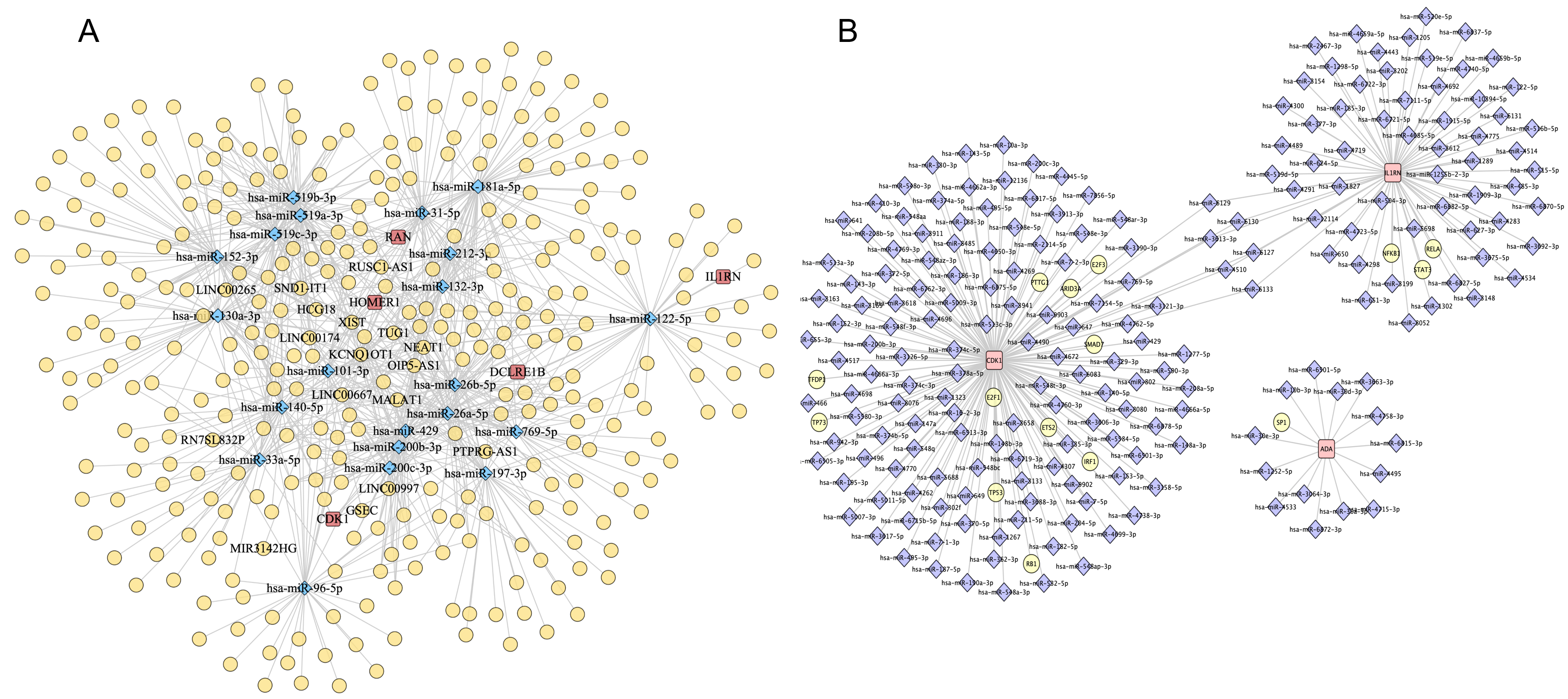

Supplement: Supplementary Figure 1 — Construction of competing endogenous RNA (ceRNA) and microRNA (miRNA)/transcription factor (TF)-gene networks. (A) ceRNA network of characteristic genes (red squares, blue diamonds, and yellow circles indicate characteristic genes, miRNAs, and long non-coding RNAs (lncRNAs) (degree ≥ 8), respectively). (B) miRNA/TF-gene network (red squares, purple diamonds, and yellow circles indicate characteristic genes, miRNAs (degree > 1), and TFs, respectively). [file Image1.tif]
